# Combined effects of entomopathogenic fungi, plant extracts, and biorational insecticides on Morocain endemic bush cricket *Eugaster spinulosa* Johansson (Orthoptera: Tettigoniidae) infesting *Opuntia* spp.

**DOI:** 10.3389/fmicb.2025.1702421

**Published:** 2025-11-18

**Authors:** Mohamed El Aalaoui, Said Rammali, Fatima Zahra Kamal, Alin Ciobica, Cristina Albert, Vasile Burlui, Bogdan Novac, Bouchaib Bencharki, Mohamed Sbaghi

**Affiliations:** 1 Department of Plant Protection, National Institute of Agricultural Research, Rabat, Morocco; 2 Human Nutrition, Bioactives and Oncogenetics Team, Faculty of Sciences, Moulay Ismail University, Meknes, Morocco; 3 Laboratory of Agro-Alimentary and Health, Faculty of Sciences and Techniques, Hassan First University of Settat, Settat, Morocco; 4 Health Care and Biology-Health Team, 2S2D Laboratory, Higher Institute of Nursing and Health Techniques of Casablanca (ISPITS), Casablanca, Morocco; 5 Department of Biology, Faculty of Biology, “Alexandru Ioan Cuza” University of Iasi, Iasi, Romania; 6 “Olga Necrasov” Center, Department of Biomedical Research, Romanian Academy, Iasi, Romania; 7 “Ioan Haulica” Institute, Apollonia University, Iasi, Romania; 8 CENEMED Platform for Interdisciplinary Research, “Grigore T. Popa” University of Medicine and Pharmacy of Iasi, Iasi, Romania; 9 Clinical Department, Apollonia University, Iasi, Romania; 10 Faculty of Medicine, “Grigore T. Popa” University of Medicine and Pharmacy of Iasi, Iasi, Romania

**Keywords:** botanical insecticides, secondary metabolites, Solanaceae and Euphorbiaceae, phytochemical toxicity, eco-friendly pest control, plant-derived biopesticides

## Abstract

**Introduction:**

*Eugaster spinulosa* Johansson (Orthoptera: Tettigoniidae) poses a serious threat to *Opuntia spp*. cultivation in Morocco, requiring effective control strategies.

**Methods:**

This study evaluated the individual and combined effects of two entomopathogenic fungi (*Alternaria destruens* (AD) and *A. murispora* (AM) at 10^10^ conidia/mL), three plant extracts (*Nicotiana glauca* (NG), *Capsicum annuum* (CA) (Solanaceae), and *Ricinus communis* (RC) (Euphorbiaceae) at 10% (w/v)), and two biorational insecticides mineral oil at 1000 cc/hl (MO) and potassium salts of fatty acids at 300 cc/hl (PFA)) on E. spinulosa under laboratory (26±2°C, 16:8 L:D photoperiod) and screenhouse conditions (26±2°C, natural light).

**Results and discussion:**

The most effective combinations (AM+CA+MO+PFA, AM+RC+MO+PFA, and AM+NG+MO+PFA) significantly reduced egg densities by week 5 to 4.85.1 eggs in the laboratory, and 8.58.9 eggs in the screenhouse, respectively, compared to 26.3 and 44.6 eggs in the untreated controls. Motile stages (nymphs and adults) were also reduced to 2.83.2 individuals in the laboratory and 4.34.6 individuals in the screenhouse, compared to 18.7 and 29.5 individuals in controls. Moderate reductions were observed with single applications of *A. murispora* (11.5 motile stages, 14.3 eggs) and mineral oil (13.6 motile stages, 16.7 eggs) under screenhouse conditions. Tween 80 showed negligible effects. All effective treatments significantly enhanced plant visual quality, with scores reaching 9.9.3 by week 5, compared to 4.1 in the control group. These findings show that combining *A. murispora, C. annuum, R. communis, N. glauca*, and biorational insecticides effectively controls *E. spinulosa* on cactus.

## 1 Introduction

The bush cricket *Eugaster spinulosa* Johansson (Orthoptera: Tettigoniidae) is endemic to Morocco and is distinguished by its large, robust, and spiny body, which serves as a defense mechanism against predators (Massa and Fontana, 2011). This species is characteristic of arid and semi-arid regions and adopts a primarily nocturnal lifestyle to reduce water loss and avoid daytime predators (Ingrisch and Köhler, 1998). *Eugaster spinulosa* has a hemimetabolous life cycle, developing through egg, nymph (five to seven instars), and adult stages without a pupal phase (Chapman, 2013). Eggs are laid in soil or plant material and hatch into nymphs, with the life cycle lasts weeks to months, depending on environmental conditions; adults emerge in favorable seasons and use stridulation to attract mates (Arias et al., 2012). Males of *E. spinulosa* are smaller and possess stridulatory forewings that produce acoustic signals (2–28 kHz) to attract females, whereas females are larger, do not produce sound, and have a prominent ovipositor for egg-laying (Arias et al., 2012). Males are more active during mating, while females remain silent and focus on selecting oviposition sites (Bleton, 1942). The reproductive biology of *E. spinulosa* is still poorly understood. Observations in Morocco show a single annual generation, nuptial gift transfer via spermatophore during mating, and a long post-mating refractory period in males (~10 days), limiting their mating opportunities (Bleton, 1942; Gwynne, 2001). In other Tettigoniidae species, spermatophore size and composition are linked to acoustic effort and environmental stress, but these trade-offs have not yet been studied in *E. spinulosa* (Arias et al., 2012).

Ecologically, *E. spinulosa* is a conspicuous ground-dwelling herbivore feeding mainly on leaves, flowers, and sometimes young shoots of both cultivated plants and wild flora (Ingrisch and Köhler, 1998; Arias et al., 2012). Although traditionally not viewed as a major pest, recent reports from Morocco (2024) have documented severe outbreaks of *E. spinulosa* on *Opuntia* species, resulting in significant defoliation and weakening of the plants. Considering the vital economic and ecological importance of *Opuntia* in arid regions, the impact of this insect could be considerable. The severity of damage seems influenced by factors such as insect population density, seasonal climate variability, and the availability of food resources (Massa and Fontana, 2011). Under climate change scenarios, with surface temperatures in North Africa rising above global averages (Majdi et al., 2022), ectothermic organisms like *E. spinulosa* face heightened risks. Individuals dwelling on exposed rocky surfaces may encounter body temperatures during midday that reach lethal thresholds (Clusella-Trullas et al., 2008). These harsh environmental conditions could disrupt their acoustic signaling, impair mating success, and ultimately threaten population sustainability.

In light of this emerging threat to *Opuntia* cultivation, it is crucial to establish management practices that are both environmentally responsible and economically feasible. Sustainable pest control options, including the use of biological agents and plant-derived extracts, are increasingly favored for their safety and effectiveness, offering alternatives that reduce dependency on synthetic chemical pesticides (Broumandnia and Rajabpour, 2020; Abbas et al., 2022; Mousavi et al., 2022). Among the biological options, plant extracts from *Ricinus communis* L. (Euphorbiaceae), *Capsicum annuum* L., and *Nicotiana glauca* Graham (Solanaceae)—all of which are widely available in Morocco—have demonstrated potent insecticidal activity against key pests such as *Dactylopius opuntiae* (Cockerell) (Hemiptera: Dactylopiidae) and *Spodoptera exigua* (Hübner) (Lepidoptera: Noctuidae) (Ramdani et al., 2021; El Aalaoui and Sbaghi, 2025). The primary bioactive compounds include ricin (a toxic protein) and ricinine (an alkaloid) in *R. communis* (Elijah and Somadina, 2020), capsaicin and dihydrocapsaicin in *C. annuum* (Zewdie and Bosland, 2001), and pyridine alkaloids such as nicotine and anabasine in *N. glauca* (Alghamdi, 2021). Although Botanical extracts are eco-friendly alternatives but face practical limits like inconsistent efficacy, fast degradation, high costs, potential phytotoxicity, slower action, and short shelf life (Ramdani et al., 2021).

The entomopathogenic potential of fungi in the genus *Alternaria* has been documented against various insect pests (Sharma and Sharma, 2021). Kaur et al. (2019) reported that larvae of *Spodoptera litura* (Fabricius) (Lepidoptera: Noctuidae) feeding on protein fractions derived from *A. destruens* AKL-3 (Fr.) had reduced growth rates (32–54%) and food consumption (19–73%), lowering their food conversion efficiency. Similarly, isolates of *Alternaria murispora* (PP264308) and *Alternaria destruens* (PP264311), obtained from *D. opuntiae* (Moroccan biotype), have demonstrated efficacy against pests such as *D. opuntiae* itself (El Aalaoui et al., 2024a), *Diaspis echinocacti* (Bouché) (Hemiptera: Diaspididae) (El Aalaoui et al., 2024b), *Phenacoccus solenopsis* (Hemiptera: Pseudococcidae) (El Aalaoui et al., 2024c), and *Cassida vittata* Vill. (Coleoptera: Chrysomelidae) (El Aalaoui and Sbaghi, 2024) under laboratory and field conditions. These characteristics position *A. destruens* and *A. murispora* as promising candidates in integrated pest management (IPM) programs, offering an environmentally sustainable alternative to chemical pesticides (Abdullah et al., 2024). Nevertheless, the relatively slow action of entomopathogenic fungi remains a challenge for their widespread adoption (Lomer et al., 2001; Pelizza et al., 2015).

Biorational insecticides have recently shown strong potential against various insect pests (Rajabpour, 2025; Kanchupati et al., 2025). Mineral oils kill by coating the insect's spiracles, causing suffocation (Helmy et al., 2012). Insecticidal soaps, made from potassium salts of fatty acids, damage cell membranes after penetrating the cuticle, leading to dehydration and death (Tsolakis and Ragusa, 2008). However, their effectiveness may decline under field conditions, and repeated use can lead to resistance (Lamsal et al., 2023).

In response to these challenges, and based on their proven efficacy and bioactive properties, the fungi (*A. murispora* and *A. destruens*), plant extracts (*N. glauca, C. annuum*, and *R. communis*), and biorational insecticides (mineral oil and potassium salts of fatty acids) were selected for this study as complementary control agents against *E. spinulosa*, providing a multi-modal strategy suitable for integrated pest management (IPM). This IPM approach, which combines entomopathogenic fungi, botanical extracts, and biorational insecticides, can enhance suppression of *E. spinulosa*, reduce dependency on synthetic chemical pesticides, delay resistance development, and promote environmentally sustainable cactus production systems. However, the combined effects of these agents against *E. spinulosa* remain largely unexplored. We hypothesize that integrating fungi, plant extracts, and biorational insecticides will result in greater pest suppression than individual treatments. Therefore, the objectives of this study were to evaluate the individual and combined effects of these agents on *E. spinulosa* under controlled laboratory and screenhouse conditions, providing critical insights for the development of effective and sustainable IPM strategies.

## 2 Material and method

### 2.1 Insect sampling and rearing

*Eugaster spinulosa* adults and nymphs were collected from *Opuntia* spp. in the Rhamna zone (Marrakech-Safi region, Morocco) (31 °33′N, 8 °34′W, and elevation 450 m). This area experiences an arid to semi-arid climate characterized by hot summers and mild winters. Average annual temperatures range from approximately 12 °C during the coolest months (December–January) to highs of 38 °C or more in summer (July–August). Annual rainfall is low, between 150 and 300 mm, mostly concentrated in the cooler season from November to March. The insects were transported to the laboratory in plastic containers (11 × 7 × 3 cm). At the National Institute of Agricultural Research (INRA) insectarium in Zemamra (32 °37′48″ N, 8 °42′0″ W; elevation 165 m), the insects were reared in aluminum cages (50 × 50 × 50 cm) covered with mesh on all sides and glass at the bottom to ensure adequate ventilation and facilitate monitoring of insect developmental stages and infestation levels. The cages were maintained under controlled conditions (25 ± 2 °C temperature, 60 ± 10% relative humidity, and a 12:12 h light-to-dark photoperiod). Fresh cladodes and fruits of *Opuntia* spp. were provided as needed to sustain the insect colony, which also served as a substrate for egg-laying. Voucher specimens of *E. spinulosa*, verified by the authors, have been archived at the Insectarium of the National Institute of Agricultural Research in Zemamra, Morocco. The insects were reared for two generations before use in experiments, and various life stages of *E. spinulosa* needed for the studies were obtained from these colony strains.

### 2.2 Botanical extracts

Plant materials of *R. communis* (seeds), *C. annuum* (fruits), and *N. glauca* (leaves) were collected from fields in Zemamra, Morocco, ensuring minimal exposure to environmental contaminants. The samples were thoroughly rinsed with distilled water and air-dried under shaded conditions for 2 weeks. Once dried to constant weight, the materials were ground into fine powder using an electric grinder (Model XYZ, Braun GmbH, Kronberg, Germany) and stored in biodegradable plastic bags (BioBag^®^, Norway) until further use. For extract preparation, 100 g of each powdered sample was mixed with 1 l of boiling distilled water. The mixture was removed from the heat source and left to infuse for 12 h at ambient temperature. After infusion, the solution was filtered through muslin cloth (Cheesecloth, Newtex Industries Inc., Pawtucket, RI, USA) and diluted with cold distilled water to a final volume of 1 l, producing a 10% (w/v) aqueous extract, following the method described by Ramdani et al. (2021). This concentration corresponds with field application rates recommended for various insects pest control (Ramdani et al., 2021; Abdelgader and Elawad, 2022; El Aalaoui and Sbaghi, 2025).

### 2.3 Fungal conidial suspension

The entomopathogenic fungi *Alternaria destruens* (NCBI GenBank Acc. No: PP264311) and *Alternaria murispora* (NCBI GenBank Acc. No: PP264308) were obtained from the INRA insectarium in Zemamra. These isolates were originally recovered from sterilized cadavers of *D. opuntiae*, the false cochineal pest of *Opuntia* spp. (Moroccan biotype) (El Aalaoui et al., 2024a). Fungal cultures were grown on potato dextrose agar (PDA), prepared with 250 g potatoes, 25 g dextrose, and 20 g agar per liter, sterilized by autoclaving at 120 °C for 20 min (Goettel et al., 2005). The PDA plates were inoculated and incubated at 26 °C in darkness for 15 days to allow sporulation. Conidia were harvested by rinsing the fungal cultures with sterile water containing 0.003% (v/v) Tween 80, then centrifuged at 5,000 rpm for 5 min. The conidial concentration was adjusted to 1.0 × 10^10^ conidia/mL using a hemocytometer, matching recommended doses for field application against *Opuntia* insects pest, including *D. opuntiae* and *Diaspis echinocacti* (Bouché) (Hemiptera: Diaspididae) (El Aalaoui et al., 2024a,b). Conidial viability was confirmed before each experiment by the method of Inglis et al. (2012), consistently exceeding 98%. Prepared suspensions were stored at 4 °C and used within 12 h to maintain potency.

### 2.4 Biorational insecticides

The biorational insecticides applied in this study included mineral oil at 1,000 cc/hL (Insecticide 101; UPL, India) and potassium salts of fatty acids at 300 cc/hL (Hamper; Gowan Crop Protection, Italy). These doses were chosen based on manufacturers' recommended field rates and supported by prior studies confirming their lack of phytotoxicity on *Opuntia* spp. and minimal harmful effects on non-target beneficial insects such as *Cryptolaemus montrouzieri* (Mulsant) (Coleoptera: Coccinellidae) (El Aalaoui et al., 2019; Aramideh and Hosseinzadeh, 2022; El Aalaoui and Sbaghi, 2025).

### 2.5 Study site and host tissue

The study was conducted under two settings: in the laboratory using the rearing cages previously described (see Section 2.1) at controlled conditions of 26 ± 2 °C, 60 ± 10% relative humidity, and a 16:8 h light-dark photoperiod; and in a screenhouse (11 × 7 × 3 m) enclosed with fine white mesh and supported by iron frames, where temperature and humidity averaged 26 ± 2 °C and 60 ± 10%, respectively, under natural light. In both setups, *Opuntia ficus-indica* (L.) Mill. plants were used as hosts. These were grown in plastic pots (33 cm in diameter, 12 cm in height) filled with a substrate composed of two-thirds fine sand and one-third peat. The plants were cultivated in an unregulated greenhouse (11 × 7 × 3 m) until they developed three to five cladodes over approximately 3 months and were watered as needed.

### 2.6 Treatments

In both laboratory and screenhouse experiments, the following treatments were applied: T1—untreated control, T2−0.003% (v/v) Tween 80 (TW), T3—*A. destruens* at 1.0 × 10^10^ conidia/mL alone (AD), T4—*A. murispora* at 1.0 × 10^10^ conidia/mL alone (AM), T5—*N. glauca* at 10% (w/v) (NG), T6—*R. communis* at 10% (w/v) (RC), T7—*C. annuum* at 10% (w/v) (CA), T8—mineral oil at 1,000 cc/hL (MO), T9—potassium salts of fatty acids at 300 cc/hL (PFA), T10—AD + NG + MO + PFA, T11—AD + RC + MO + PFA, T12—AD + CA + MO + PFA, T13—AM + NG + MO + PFA, T14—AM + RC + MO + PFA, and T15—AM + CA + MO + PFA. The combination of both fungal species was not included, as it was not the objective of this study. All plant extracts and biorational insecticides were applied at a rate of 30 mL per plant using a 1.5 l garden pressure sprayer (Mesto Spritzenfabrik Ernst Stockburger GmbH, Germany). Prior to treatment application, each *O. ficus-indica* plant was infested with 20 adult *E. spinulosa* (approximately 10 females and 10 males) using a fine brush and allowed to establish for 15 days. Treatments were applied directly to both the plants and the insects. In the screenhouse, to prevent cross-contamination, each plant was enclosed in a ventilated cylindrical cage made of sealed transparent plastic film, with the top covered by coarse mesh organdy fabric to allow airflow. In both experiments, treatments were arranged in a randomized complete block design with three replicates per treatment, each replicate consisting of three *E. spinulosa*-infested *O. ficus-indica* plants, totaling 135 plants per experiment. The entire experiment was conducted twice at different times.

The experimental design focused on evaluating the potential synergistic effects of integrating a single fungal species with one plant extract and the two biorational insecticides (mineral oil and potassium salts of fatty acids). Binary or ternary combinations were not included because the primary objective was to test the most practical and effective multi-modal treatment under controlled conditions, reflecting a realistic integrated pest management scenario. This approach allows for assessing whether combining a single entomopathogenic fungus with a botanical extract and biorational insecticides provides enhanced suppression of *E. spinulosa* compared with individual treatments, while maintaining manageable experimental complexity.

### 2.7 Assessment of the effectiveness of treatments

In both laboratory and screenhouse experiments, the initial density of *E. spinulosa* was assessed across all treatments 10 days after infestation, prior to treatment application. The numbers of motile stages (nymphs and adults) and eggs of *E. spinulosa* were recorded using a 2.75 × hand lens. Treatment efficacy was monitored starting 1 week after application and continued weekly for five consecutive weeks, with counts of *E. spinulosa* motile stages and eggs recorded at each interval. Additionally, plant damage caused by *E. spinulosa* was evaluated weekly after treatment using a visual scale from 0 to 10, where 0 corresponds to a dead plant, 5 indicates moderate quality with acceptable color and form and minimal chlorosis or necrosis, and 10 represents excellent condition with healthy appearance, ideal color, and form. This scale was originally described by Gettys et al. (2021) for evaluating plant quality in aquatic plants and was adapted for *Opuntia* plants in the present study. To ensure its suitability, preliminary observations were conducted on infested *Opuntia* to verify that the scoring categories accurately reflected the severity of damage caused by *E. spinulosa*. The criteria were adjusted to account for *Opuntia*-specific traits such as pad and stem appearance, chlorosis, necrosis, and overall plant vigor. Multiple observers assessed a subset of plants to confirm consistency and reproducibility of the scoring. This adapted scale provided a simple, reliable, and standardized method for assessing plant health and treatment efficacy across all experimental treatments. Similar methods have been applied to assess the effects of herbicides, salt stress, and other stressors on plant health (Gettys and Haller, 2012; Tootoonchi et al., 2020).

### 2.8 Statistical analyses

Statistical analyses were conducted using R software version 4.3.2 to evaluate the effectiveness of the 15 treatments on *E. spinulosa* populations and the visual quality of *O. ficus-indica* plants. Prior to analysis, all count data (e.g., motile stages and eggs of *E. spinulosa*) were tested for normality using the Shapiro-Wilk test and for homogeneity of variances using Levene's test. When assumptions were not met, data were log-transformed using the formula log_10_(*x* + 1). Treatment effects on the number of motile *E. spinulosa* stages, eggs, and visual plant quality scores were analyzed using one-way ANOVA followed by Student-Newman-Keuls (SNK) *post-hoc* tests to identify significant differences among treatments. Separate analyses were conducted for data obtained from the laboratory and screenhouse experiments. For repeated weekly measures of insect density and plant damage over 5 weeks, data were analyzed using repeated measures ANOVA, with treatment as the between-subject factor and time as the within-subject factor. All results were considered statistically significant at α = 0.05. Data were graphically visualized to illustrate temporal dynamics in pest suppression and plant health across treatments under both laboratory and screenhouse conditions.

## 3 Results

### 3.1 Laboratory trials

#### 3.1.1 Efficacy of treatments on Eugaster spinulosa eggs

The results showed no significant effect of the treatments on *E. spinulosa* egg counts before treatment application (Table 1). In week 1 post-treatment, there was a significant reduction in egg counts for several treatments compared to the control and Tween 80. By week 2, the treatment effect became more pronounced, with AM + CA + MO + PFA, AM + RC + MO + PFA, and AM + NG + MO + PFA treatments showing the lowest egg densities. In week 3, the strong treatment effect highlighted AM + CA + MO + PFA, AM + NG + MO + PFA, and AM + RC + MO + PFA treatments as the most effective in reducing egg counts. Week 4 results continued to show a significant treatment effect, with AM + NG + MO + PFA, AM + RC + MO + PFA, and AM + CA + MO + PFA treatments performing significantly better than the other treatments. In week 5 post-treatment, the highly significant treatment effect was evident, with AM + CA + MO + PFA (4.8 eggs), AM + NG + MO + PFA (4.8 eggs), and AM + RC + MO + PFA (4.9 eggs) treatments emerging as the most effective treatments. Additionally, prolonged exposure significantly enhanced treatment efficacy in reducing *E. spinulosa* egg densities (Table 1). AD treatment showed a significant reduction from 27.2 to 15.1 eggs [*F*_(5, 48)_ = 114.2, *P* < 2.0 × 10^−16^], AM treatment from 27.9 to 11.4 eggs [*F*_(5, 48)_ = 323.1, *P* < 2.0 × 10^−16^], NG treatment from 27.2 to 13.9 eggs [*F*_(5, 48)_ = 214.6, *P* < 2.0 × 10^−16^], RC treatment from 27.5 to 13.3 eggs [*F*_(5, 48)_ = 248.9, *P* < 2.0 × 10^−16^], T7 (CA) from 27.2 to 13.3 eggs [*F*_(5, 48)_ = 200.8, *P* < 2.0 × 10^−16^], MO treatment from 26.2 to 12.5 eggs [*F*_(5, 48)_ = 225.3, *P* < 2.0 × 10^−16^], PFA treatment from 26.5 to 12.0 eggs [*F*_(5, 48)_ = 561.3, *P* < 2.0 × 10^−16^], AD + NG + MO + PFA treatment from 26.9 to 5.5 eggs [*F*_(5, 48)_ = 2620.0, *P* < 2.0 × 10^−16^], AD + RC + MO + PFA treatment from 27.4 to 5.5 eggs [*F*_(5, 48)_ = 3513.0, *P* < 2.0 × 10^−16^], and AD + CA + MO + PFA treatment from 27.0 to 5.4 eggs [*F*_(5, 48)_ = 2249.0, *P* < 2.0 × 10^−16^]. The most pronounced reductions were recorded in AM + NG + MO + PFA [*F*_(5, 48)_ = 804.8, *P* < 2.0 × 10^−16^], AM + RC + MO + PFA [*F*_(5, 48)_ = 981.4, *P* < 2.0 × 10^−16^], and AM + CA + MO + PFA [*F*_(5, 48)_ = 763.7, *P* < 2.0 × 10^−16^] treatments, with egg counts dropping from ~27.7 to below 5 eggs.

**Table 1:** Effect of different treatments on densities of eggs of *Eugaster spinulosa* under laboratory conditions (26 ± 2 °C, 60 ± 10% relative humidity, and a 12:12 h l: D photoperiod).

**Treatment**	**Density of eggs (Mean** ±**SE)**					
	**Before treatment**	**Week 1**	**Week 2**	**Week 3**	**Week 4**	**Week 5**
T1	27.7 ± 0.4 Aa	26.6 ± 0.3 Aa	26.8 ± 0.6 Aa	26.9 ± 0.3 Aa	26.7 ± 0.2 Aa	26.3 ± 0.3 Aa
T2	27.3 ± 0.3 Aa	25.5 ± 0.5 Ab	25.2 ± 0.3 Bb	25.0 ± 0.2 Bb	25.1 ± 0.4 Bb	25.2 ± 0.3 Ab
T3	27.2 ± 0.4 Aa	20.2 ± 0.5 Bb	19.1 ± 0.4 Cc	17.6 ± 0.3 Cd	16.9 ± 0.3 Cd	15.1 ± 0.2 Be
T4	27.9 ± 0.5 Aa	16.9 ± 0.3 Eb	15.0 ± 0.3 Ec	13.4 ± 0.1 Gd	12.2 ± 0.2 Fe	11.4 ± 0.2f
T5	27.2 ± 0.3 Aa	20.7 ± 0.4 Bb	18.8 ± 0.2 Cc	16.9 ± 0.3 CDd	16.1 ± 0.2 Cd	13.9 ± 0.2 Ce
T6	27.5 ± 0.5 Aa	19.6 ± 0.3 BCb	18.0 ± 0.2 Cc	16.0 ± 0.1 DEd	14.7 ± 0.2 De	13.3 ± 0.2 CDf
T7	27.2 ± 0.5 Aa	19.8 ± 0.5 BCb	18.1 ± 0.3 Cc	16.0 ± 0.3 DEd	14.5 ± 0.2 DEe	13.3 ± 0.2 Cf
T8	26.2 ± 0.4 Aa	19.1 ± 0.4 CDb	16.6 ± 0.2 Dc	14.7 ± 0.2 Fd	13.8 ± 0.2 DEe	12.5 ± 0.2 DEf
T9	26.5 ± 0.6 Aa	18.2 ± 0.3 Db	16.7 ± 0.3 Dc	15.0 ± 0.3 EFd	13.6 ± 0.2 Ee	12.0 ± 0.2 EFf
T10	26.9 ± 0.4 Aa	13.0 ± 0.2 Fb	9.5 ± 0.2 Fc	7.5 ± 0.2 Hd	6.6 ± 0.1 Ge	5.5 ± 0.1 Gf
T11	27.4 ± 0.4 Aa	12.7 ± 0.2 FGb	9.8 ± 0.3 Fc	7.8 ± 0.1 Hd	6.5 ± 0.1 Ge	5.5 ± 0.1 Gf
T12	27.0 ± 0.6 Aa	12.5 ± 0.2 FGb	9.6 ± 0.1 Fc	7.9 ± 0.1 Hd	6.4 ± 0.1 Ge	5.4 ± 0.1 Gf
T13	27.7 ± 0.4 Aa	12.1 ± 0.2 Gb	9.0 ± 0.1 Gc	7.0 ± 0.2 Id	5.8 ± 0.1 He	4.8 ± 0.1 Hf
T14	27.9 ± 0.5 Aa	12.4 ± 0.2 FGb	9.3 ± 0.2 Gc	6.9 ± 0.2 Id	5.7 ± 0.1 He	4.9 ± 0.1 Hf
T15	27.1 ± 0.6 Aa	12.1 ± 0.1 Gb	8.8 ± 0.1 Gc	7.0 ± 0.1 Id	5.7 ± 0.1 He	4.8 ± 0.1 Hf
Statistical analysis	*F*_14, 120_ = 1.2, *P* = 0.302	*F*_14, 120_ = 233.2, *P* < 2.0 × 10^−16^	*F*_14, 120_ = 461.4, *P* < 2.0 × 10^−16^	*F*_14, 120_ = 573.2, *P* < 2.0 × 10^−16^	*F*_14, 120_ = 653.7, *P* < 2.0 × 10^−16^	*F*_14, 120_ = 821.0, *P* < 2.0 × 10^−16^

Means followed by the same capital letter within a column are not significantly different (based on the Student-Newman-Keuls test, α = 0.05). Means followed by the same lowercase letter within a row are not significantly different (based on the Student-Newman-Keuls test, α = 0.05). T1 = untreated control; T2 = 0.003% (v/v) Tween 80; T3 = Alternaria destruens at 1.0 × 10^10^ conidia/mL alone (AD); T4 = Alternaria murispora at 1.0 × 10^10^ conidia/mL alone (AM); T5 = Nicotiana glauca at 10% (NG); T6 = Ricinus communis at 10% (RC); T7 = Capsicum annuum at 10% (CA); T8 = mineral oil at 1,000 cc/hL (MO); T9 = potassium salts of fatty acids at 300 cc/hL (PFA); T10 = AD + NG + MO + PFA; T11 = AD + RC + MO + PFA; T12 = AD + CA + MO + PFA; T13 = AM + NG + MO + PFA; T14 = AM + RC + MO + PFA; and T15 = AM + CA + MO + PFA.

#### 3.1.2 Efficacy of treatments on Eugaster spinulosa motile stages

The results showed no significant effect of the treatments on *E. spinulosa* motile stages before treatment application (Table 2). In week 1 post-treatment, there was a highly significant reduction in motile stage densities for several treatments compared to the control and Tween 80. By week 2, the treatment effect was more pronounced, with treatments AM + NG + MO + PFA, AM + RC + MO + PFA, and AM + CA + MO + PFA showing the lowest motile stage densities. In week 3, the treatment effect remained strong, with AM + NG + MO + PFA, AM + RC + MO + PFA, and AM + CA + MO + PFA treatments being the most effective. Week 4 results showed continued significant treatment effects, with AM + NG + MO + PFA, AM + RC + MO + PFA, and AM + CA + MO + PFA treatments performing significantly better than the other tested treatments. In week 5 post-treatment, the treatment effect remained highly significant, with AM + NG + MO + PFA (2.8 insects), AM + RC + MO + PFA (2.8 insects), and AM + CA + MO + PFA (2.9 insects) treatments emerging as the most effective. Additionally, prolonged exposure significantly enhanced the efficacy of all treatments in reducing *E. spinulosa* motile stages (Table 2). AD treatment showed a significant reduction from 17.1 motile stages before treatment application 0 to 7.1 at week 5 [*F*_(5, 48)_ = 339.0, *P* < 2.0 × 10^−16^], AM treatment from 17.3 to 5.1 1 [*F*_(5, 48)_ = 487.5, *P* < 2.0 × 10^−16^], NG treatment from 16.9 to 7.9 [*F*_(5, 48)_ = 255.6, *P* < 2.0 × 10^−16^], RC treatment from 16.5 to 7.5 [*F*_(5, 48)_ = 472.7, *P* < 2.0 × 10^−16^], CA treatment from 17.1 to 7.3 [*F*_(5, 48)_ = 561.3, *P* < 2.0 × 10^−16^], MO treatment from 17.2 to 5.7 [*F*_(5, 48)_ = 576.7, *P* < 2.0 × 10^−16^], PFA treatment from 17.0 to 5.7 [*F*_(5, 48)_ = 561.3, *P* < 2.0 × 10^−16^], AD + NG + MO + PFA treatment from 17.2 to 3.1 [*F*_(5, 48)_ = 2620.0, *P* < 2.0 × 10^−16^], AD + RC + MO + PFA treatment from 16.8 to 3.2 [*F*_(5, 48)_ = 3513.0, *P* < 2.0 × 10^−16^], and AD + CA + MO + PFA treatment from 16.6 to 3.2 [*F*_(5, 48)_ = 2249.0, *P* < 2.0 × 10^−16^]. The most pronounced reductions were recorded in AM + NG + MO + PFA [*F*_(5, 48)_ = 854.9, *P* < 2.0 × 10^−16^], AM + RC + MO + PFA [*F*_(5, 48)_ = 1,748, *P* < 2.0 × 10^−16^], and AM + CA + MO + PFA [*F*_(5, 48)_ = 1,839, *P* < 2.0 × 10^−16^] treatments, where motile stage densities dropped from approximately 17.4 to below 3 motile stages by week 5.

**Table 2:** Effect of different treatments on motile stages (nymphs and adults) of *Eugaster spinulosa* under laboratory conditions (26 ± 2 °C, 60 ± 10% relative humidity, and a 12:12 h l: D photoperiod).

**Treatment**	**Density of eggs (Mean** ±**SE)**					
	**Before treatment**	**Week 1**	**Week 2**	**Week 3**	**Week 4**	**Week 5**
T1	17.6 ± 0.3 Aa	17.2 ± 0.4 Aa	17.4 ± 0.2 Aa	17.1 ± 0.2 Aa	16.9 ± 0.1 Aa	16.8 ± 0.3 Aa
T2	17.1 ± 0.3 Aa	16.2 ± 0.2 Bb	16.3 ± 0.3 Bb	15.9 ± 0.3 Bbc	15.9 ± 0.2 Bbc	15.2 ± 0.1 Bc
T3	17.1 ± 0.2 Aa	12.4 ± 0.3 Cb	11.2 ± 0.2 Dc	9.9 ± 0.1 Cd	8.5 ± 0.2 De	7.1 ± 0.1 Ef
T4	17.3 ± 0.2 Aa	9.8 ± 0.3 Eb	8.9 ± 0.2 Fc	7.2 ± 0.1 Ed	6.1 ± 0.1 Fe	5.1 ± 0.1 Gf
T5	16.9 ± 0.3 Aa	13.1 ± 0.1 Cb	12.2 ± 0.2 Cc	10.3 ± 0.2 Cd	9.2 ± 0.2 Ce	7.9 ± 0.1 Cf
T6	16.5 ± 0.2 Aa	13.1 ± 0.2 Cb	11.8 ± 0.1 Cc	10.1 ± 0.2 Cd	8.9 ± 0.1 CDe	7.5 ± 0.1 CDf
T7	17.1 ± 0.1 Aa	13.2 ± 0.2 Cb	11.3 ± 0.1 Dc	9.8 ± 0.1 Cd	8.5 ± 0.2 De	7.3 ± 0.1 DEf
T8	17.2 ± 0.2 Aa	11.4 ± 0.2 Db	9.9 ± 0.1 Ec	8.5 ± 0.1 Dd	6.9 ± 0.1 Ee	5.7 ± 0.1 Ff
T9	16.9 ± 0.2 Aa	11.5 ± 0.1 Db	9.5 ± 0.2 Ec	8.6 ± 0.2 Dd	6.7 ± 0.1 Ee	5.7 ± 0.1 Ff
T10	17.2 ± 0.2 Aa	6.5 ± 0.1 FGb	5.3 ± 0.1 Gc	4.6 ± 0.1 Fd	3.6 ± 0.1 GHe	3.1 ± 0.1 Hf
T11	16.8 ± 0.3 Aa	6.8 ± 0.1 Fb	5.4 ± 0.1 Gc	4.5 ± 0.1 FGd	3.5 ± 0.1 GHIe	3.2 ± 0.1 Hf
T12	16.6 ± 0.2 Aa	6.5 ± 0.1 FGb	5.3 ± 0.1 Gc	4.3 ± 0.1 GHd	3.7 ± 0.1 Ge	3.2 ± 0.1 Hf
T13	17.4 ± 0.3 Aa	6.0 ± 0.2 Hb	4.8 ± 0.1 Hc	4.2 ± 0.1 Hd	3.3 ± 0.1 HIe	2.8 ± 0.1 If
T14	17.3 ± 0.2 Aa	6.4 ± 0.1 GHb	4.9 ± 0.1 Hc	4.2 ± 0.1 Hd	3.3 ± 0.1 Ie	2.8 ± 0.0 If
T15	17.8 ± 0.2 Aa	6.2 ± 0.1 GHb	5.0 ± 0.1 Hc	4.2 ± 0.1 Hd	3.4 ± 0.1 HIe	2.9 ± 0.0 If
Statistical analysis	*F*_14, 120_ = 1.4, *P* = 0.618	*F*_14, 120_ = 451.3, *P* < 2.0 × 10^−16^	*F*_14, 120_ = 720.2, *P* < 2.0 × 10^−16^	*F*_14, 120_ = 1012.0, *P* < 2.0 × 10^−16^	*F*_14, 120_ = 981.5, *P* < 2.0 × 10^−16^	*F*_14, 120_ = 1082.0, *P* < 2.0 × 10^−16^

Means followed by the same capital letter within a column are not significantly different (based on the Student-Newman-Keuls test, α = 0.05). Means followed by the same lowercase letter within a row are not significantly different (based on the Student-Newman-Keuls test, α = 0.05). T1 = untreated control; T2 = 0.003% (v/v) Tween 80; T3 = Alternaria destruens at 1.0 × 10^10^ conidia/mL alone (AD); T4 = Alternaria murispora at 1.0 × 10^10^ conidia/mL alone (AM); T5 = Nicotiana glauca at 10% (NG); T6 = Ricinus communis at 10% (RC); T7 = Capsicum annuum at 10% (CA); T8 = mineral oil at 1,000 cc/hL (MO); T9 = potassium salts of fatty acids at 300 cc/hL (PFA); T10 = AD + NG + MO + PFA; T11 = AD + RC + MO + PFA; T12 = AD + CA + MO + PFA; T13 = AM + NG + MO + PFA; T14 = AM + RC + MO + PFA; and T15 = AM + CA + MO + PFA.

#### 3.1.3 Effect of treatments on treated plant visual quality

**Figure 1:**
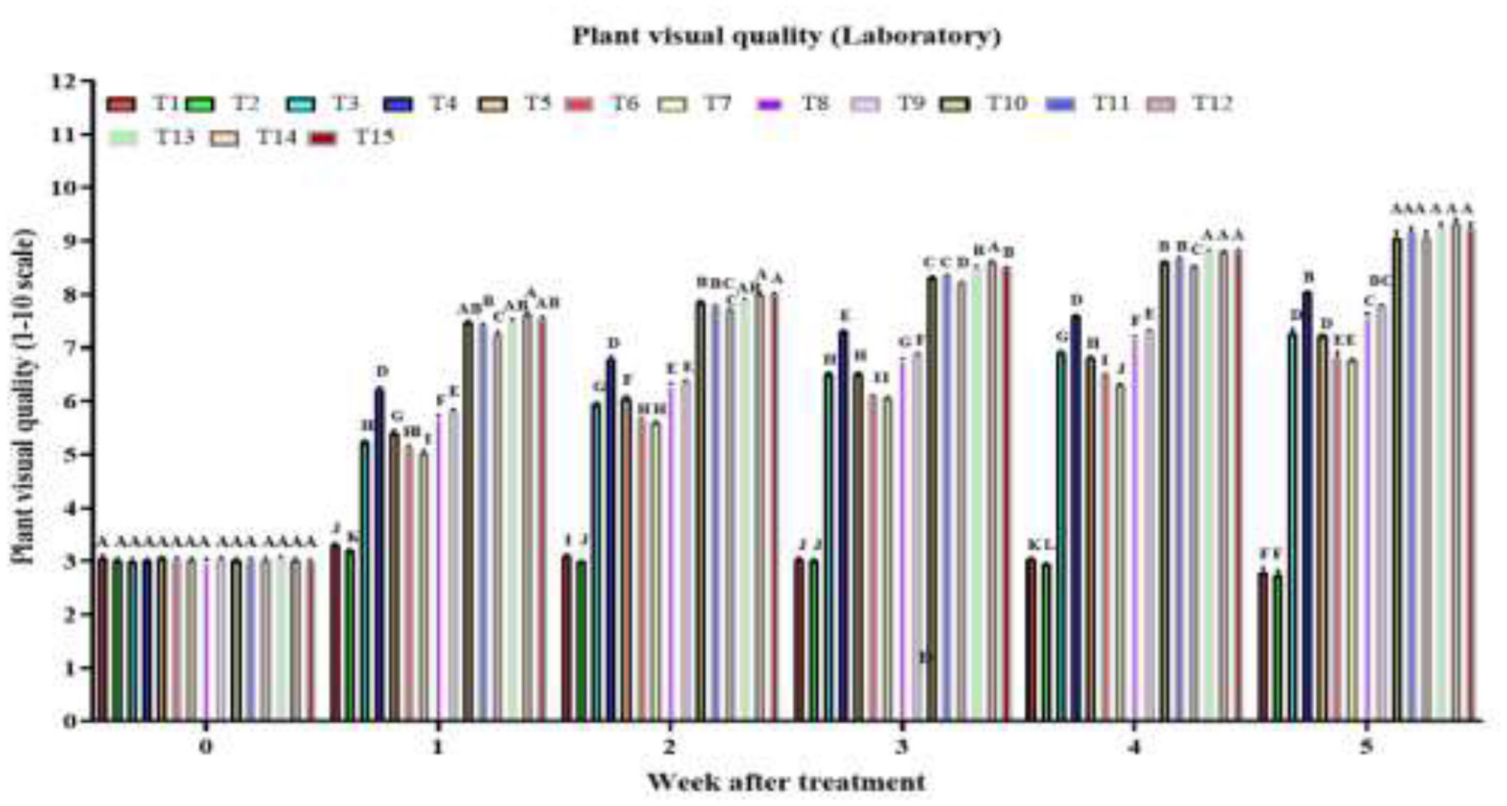
Visual quality of *O. ficus-indica* plants after spray application of untreated control (T1), 0.003% (v/v) Tween 80 (T2), *Alternaria destruens* at 1.0 × 10^10^ conidia/mL alone (T3), *Alternaria murispora* at 1.0 × 10^10^ conidia/mL alone (T4), *Nicotiana glauca* at 10% (T5), *Ricinus communis* at 10% (T6), *Capsicum annuum* at 10% (T7), mineral oil at 1,000 cc/hL (T8), potassium salts of fatty acids at 300 cc/hL (T9), AD + NG + MO + PFA (T10), AD + RC + MO + PFA (T11), AD + CA + MO + PFA (T12), AM + NG + MO + PFA (T13); AM + RC + MO + PFA (T14), and AM + CA + MO + PFA (T15).Visual quality is rated on a scale from 0 to 10, where 0 represents dead; 5 denotes fair quality with acceptable form and color, minimal chlorosis or necrosis; and 10 indicates excellent quality, healthy and robust with optimal color and form. Different letters above bars indicate statistical differences (based on the Student-Newman-Keuls test, α = 0.05). The order of treatments in the figure corresponds exactly to the order listed here, from left to right, to facilitate tracking of each treatment's data.

By week 1, there was a highly significant treatment effect on plant visual quality score [*F*_(14, 120)_ = 1479.0, *P* < 2 × 10^−16^], with combined treatments AM + RC + MO + PFA, AM + CA + MO + PFA, AM + NG + MO + PFA and AD + NG + MO + PFA showing the highest visual quality scores (Figure 1). In week 2, the treatment effect remained highly significant [*F*_(14, 120)_ = 3604.0, *P* < 2 × 10^−16^], with the same combined treatments maintaining superior scores. The strong treatment effect persisted in week 3 [*F*_(14, 120)_ = 10000.0, *P* < 2 × 10^−16^], with AM + RC + MO + PFA treatment being the most effective. By week 4, the significant effect continued [*F*_(14, 120)_ = 11,967, *P* < 2 × 10^−16^], with AM + NG + MO + PFA, AM + CA + MO + PFA and AM + RC + MO + PFA treatments achieving the highest scores. In week 5, the highly significant treatment effect [*F*_(14, 120)_ = 632.1, *P* < 2 × 10^−16^] highlighted the tested combined treatments AM + RC + MO + PFA (9.3), AM + NG + MO + PFA (9.2), AM + CA + MO + PFA (9.2), AD + RC + MO + PFA (9.2), AD + NG + MO + PFA (9.1), and AD + CA + MO + PFA (9.1) as the most effective. The control treatment showed a decline in visual quality score from 3.1 before treatment to 2.8 at week 5 post treatment [*F*_(5, 48)_ = 8.9, *P* = 4.4 × 10^−6^]. Tween 80 treatment decreased the visual quality score from 3.0 to 2.7 [*F*_(5, 48)_ = 9.1, *P* = 3.7 × 10^−6^]. The AD treatment improved the visual quality score from 3.0 to 7.3 [*F*_(5, 48)_ = 1047.0, *P* = 2.0 × 10^−16^]. The AM treatment scores increased from 3.0 to 8.0 [*F*_(5, 48)_ = 3836.0, *P* < 2.0 × 10^−16^], NG treatment scores increased from 3.1 to 7.2 [*F*_(5, 48)_ = 2,105, *P* < 2.0 × 10^−16^], RC treatment from 3.0 to 6.8 [*F*_(5, 48)_ = 891.8, *P* < 2.0 × 10^−16^], CA treatment from 3.0 to 6.8 [*F*_(5, 48)_ = 1464.0, *P* < 2.0 × 10^−16^], MO treatment from 3.0 to 7.6 [*F*_(5, 48)_ = 2281.0, *P* < 2.0 × 10^−16^], PFA treatment from 3.0 to 7.8 [*F*_(5, 48)_ = 2488.0, *P* < 2.0 × 10^−16^], AD + NG + MO + PFA treatment from 3.0 to 9.1 [*F*_(5, 48)_ = 2620.0, *P* < 2.0 × 10^−16^], AD + RC + MO + PFA treatment from 3.0 to 9.2 [*F*_(5, 48)_ = 3513.0, *P* < 2.0 × 10^−16^], and AD + CA + MO + PFA treatment from 3.0 to 9.1 [*F*_(5, 48)_ = 2249.0, *P* < 2.0 × 10^−16^]. AM + NG + MO + PFA [*F*_(5, 48)_ = 3413.0, *P* < 2.0 × 10^−16^], AM + RC + MO + PFA [*F*_(5, 48)_ = 3186.0, *P* < 2.0 × 10^−16^], and AM + CA + MO + PFA [*F*_(5, 48)_ = 3262.0, *P* < 2.0 × 10^−16^] treatments, improved the visual quality score from 3.0 to 9.2, 9.3, and 9.2, respectively.

### 3.2 Screenhouse trials

#### 3.2.1 Efficacy of treatments on Eugaster spinulosa eggs

Prior to treatment application, there were no statistically significant differences in egg densities across treatments (Table 3). By week 1 post-treatment, a strong treatment effect emerged, with the AM + CA + MO + PFA, AM + RC + MO + PFA, and AM + NG + MO + PFA treatments achieving the lowest egg counts. The treatment effect became more pronounced by week 2, with AM + NG + MO + PFA, AM + RC + MO + PFA, and AM + CA + MO + PFA treatments showing significant reductions relative to both the control and single-component treatments. In week 3, this trend persisted. By week 4, egg densities in the combination treatments continued to decline, with AM + NG + MO + PFA, AM + RC + MO + PFA, and AM + CA + MO + PFA treatments again emerging as the most effective. At week 5, the treatment effect remained highly significant, with AM + NG + MO + PFA and AM + CA + MO + PFA reducing egg counts to 8.5 eggs—representing over 80% reduction compared to the untreated control (44.6 eggs). Furthermore, within-treatment comparisons corresponding to exposure time confirmed significant temporal declines in egg densities across most treatments (Table 3). The most pronounced reductions occurred in combined treatments. In the AM + CA + MO + PFA treatment, egg density declined from 45.7 eggs before treatment to 8.5 eggs at week 5 [*F*_(5, 48)_ = 232,533.0, *P* < 2.0 × 10^−16^]. Similarly, significant reductions were observed in AM + NG + MO + PFA (from 45.7 to 8.5 eggs) [*F*_(5, 48)_ = 162,375.0, *P* < 2.0 × 10^−16^], AM + RC + MO + PFA (from 45.7 to 8.6 eggs) [*F*_(5, 48)_ = 247,324.0, *P* < 2.0 × 10^−16^], AD + CA + MO + PFA (from 45.5 to 9.7 eggs) [*F*_(5, 48)_ = 216,874.0, *P* < 2.0 × 10^−16^], AD + RC + MO + PFA (from 45.5 to 9.9 eggs) [*F*_(5, 48)_ = 213,461.0, *P* < 2.0 × 10^−16^], and AD + NG + MO + PFA (from 45.5 to 10.2 eggs) [*F*_(5, 48)_ = 184,954.0, *P* < 2.0 × 10^−16^]. Moderate but still significant reductions were also observed in treatments with single or fewer components. AD alone reduced egg density from 45.1 to 26.5 eggs [*F*_(5, 48)_ = 59,586.0, *P* < 2.0 × 10^−16^]; AM alone from 45.7 to 19.6 eggs [*F*_(5, 48)_ = 91,067.0, *P* < 2.0 × 10^−16^]; NG alone from 45.3 to 23.8 eggs [*F*_(5, 48)_ = 115,141.0, *P* < 2.0 × 10^−16^]; RC alone from 45.6 to 24.9 eggs [*F*_(5, 48)_ = 101,767.0, *P* < 2.0 × 10^−16^]; CA alone from 45.8 to 22.7 eggs [*F*_(5, 48)_ = 112,508.0, *P* < 2.0 × 10^−16^]; MO alone from 45.5 to 20.4 eggs [*F*_(5, 48)_ = 82,998.0, *P* < 2.0 × 10^−16^]; and PFA alone from 45.5 to 18.3 eggs [*F*_(5, 48)_ = 84,493.0, *P* < 2.0 × 10^−16^]. In contrast, egg densities in the untreated control remained nearly constant (45.5–45.1), while Tween 80 caused only a slight decline (45.5–41.3), confirming its negligible effect.

**Table 3:** Effect of different treatments on densities of eggs of *Eugaster spinulosa* under screehouse conditions (26 ± 2 °C, 60 ± 10% relative humidity, under natural light).

**Treatment**	**Density of eggs (Mean** ±**SE)**					
	**Before treatment**	**Week 1**	**Week 2**	**Week 3**	**Week 4**	**Week 5**
T1	45.9 ± 0.6 Aa	44.3 ± 0.4 Ab	45.1 ± 0.2 Ac	44.9 ± 0.2 Ad	44.7 ± 0.2 Ae	44.6 ± 0.2 Af
T2	45.7 ± 0.1 Aa	43.0 ± 0.3 Bb	42.4 ± 0.2 Bc	42.2 ± 0.2 Bd	42.0 ± 0.2 Be	41.8 ± 0.2 Bf
T3	45.1 ± 0.5 Aa	36.1 ± 0.3 Cb	33.2 ± 0.2 Cc	30.2 ± 0.2 Cd	28.2 ± 0.2 Ce	26.5 ± 0.1 Cf
T4	45.7 ± 0.5 Aa	28.8 ± 0.4 Ib	25.4 ± 0.2 Ic	23.4 ± 0.2 Id	21.6 ± 0.2 Ie	19.6 ± 0.2 If
T5	45.3 ± 0.4 Aa	35.0 ± 0.2 Db	31.9 ± 0.2 Dc	28.7 ± 0.2 Dd	26.1 ± 0.1 De	23.8 ± 0.1 Df
T6	45.4 ± 0.4 Aa	33.7 ± 0.3 Eb	30.7 ± 0.2 Ec	27.5 ± 0.2 Ed	25.1 ± 0.2 Ee	23.0 ± 0.2 Ef
T7	45.2 ± 0.2 Aa	33.2 ± 0.3 Fb	30.1 ± 0.2 Fc	27.1 ± 0.2 Fd	24.8 ± 0.2 Fe	22.8 ± 0.1 Ff
T8	45.2 ± 0.6 Aa	31.4 ± 0.3 Gb	28.4 ± 0.2 Gc	25.7 ± 0.2 Gd	23.4 ± 0.1 Ge	21.4 ± 0.2 Gf
T9	45.3 ± 0.6 Aa	30.6 ± 0.3 Hb	27.7 ± 0.3 Hc	25.4 ± 0.2 Hd	23.3 ± 0.2 He	21.0 ± 0.2 Hf
T10	45.7 ± 0.3 Aa	21.7 ± 0.2 Kb	17.1 ± 0.2 Jc	13.3 ± 0.2 Kd	11.3 ± 0.2 Je	10.1 ± 0.2 Jf
T11	45.5 ± 0.2 Aa	21.9 ± 0.2 Jb	16.9 ± 0.2 Kc	13.4 ± 0.2 Jd	11.3 ± 0.1 Je	9.9 ± 0.2 Kf
T12	45.5 ± 0.3 Aa	21.3 ± 0.2 Ib	16.5 ± 0.2 Lc	13.1 ± 0.2 Ld	10.9 ± 0.2 Ke	9.7 ± 0.1 Lf
T13	45.7 ± 0.2 Aa	20.5 ± 0.4 Nb	15.4 ± 0.2 Mc	12.0 ± 0.2 Od	9.8 ± 0.2 Ne	8.5 ± 0.2 Nf
T14	45.7 ± 0.3 Aa	20.6 ± 0.3 Mb	15.3 ± 0.2 Nc	12.1 ± 0.2 Md	9.9 ± 0.1 Le	8.6 ± 0.1 Mf
T15	45.7 ± 0.2 Aa	20.5 ± 0.2 MNb	15.4 ± 0.2 Mc	12.1 ± 0.2 Nd	9.9 ± 0.1 Me	8.5 ± 0.1 Nf
Statistical analysis	*F*_14, 120_ = 26.9, *P* = 0.216	*F*_14, 120_ = 62955.0, *P* < 2.0 × 10^−16^	*F*_14, 120_ = 178249.0, *P* < 2.0 × 10^−16^	*F*_14, 120_ = 200814.0, *P* < 2.0 × 10^−16^	*F*_14, 120_ = 260550.0, *P* < 2.0 × 10^−16^	*F*_14, 120_ = 248579.0, *P* < 2.0 × 10^−16^

Means followed by the same capital letter within a column are not significantly different (based on the Student-Newman-Keuls test, α = 0.05). Means followed by the same lowercase letter within a row are not significantly different (based on the Student-Newman-Keuls test, α = 0.05). T1 = untreated control; T2 = 0.003% (v/v) Tween 80; T3 = Alternaria destruens at 1.0 × 10^10^ conidia/mL alone (AD); T4 = Alternaria murispora at 1.0 × 10^10^ conidia/mL alone (AM); T5 = Nicotiana glauca at 10% (NG); T6 = Ricinus communis at 10% (RC); T7 = Capsicum annuum at 10% (CA); T8 = mineral oil at 1,000 cc/hL (MO); T9 = potassium salts of fatty acids at 300 cc/hL (PFA); T10 = AD + NG + MO + PFA; T11 = AD + RC + MO + PFA; T12 = AD + CA + MO + PFA; T13 = AM + NG + MO + PFA; T14 = AM + RC + MO + PFA; and T15 = AM + CA + MO + PFA.

#### 3.2.2 Efficacy of treatments on Eugaster spinulosa motile stages

Prior to treatment application, no statistically significant differences in motile stages densities were observed across treatments (Table 4). By week 1, however, a strong treatment effect emerged. The greatest reductions occurred with combined treatments. By week 2, effects were significant, with AM + RC + MO + PFA treatment showing the lowest motile stage density. This trend persisted in week 3 and week 4. At week 5, the treatment effect remained highly significant, with AM + NG + MO + PFA, AM + RC + MO + PFA, and AM + CA + MO + PFA treatments reducing motile stages densities to 4.4, 4.3, and 4.4 motile stages respectively—representing an 83–84% reduction compared to the untreated control (25.4 motile stages). Within-treatment comparisons across time confirmed significant temporal declines, with motile stages counts decreasing from 26.0 to 4.4 eggs in AM + CA + MO + PFA treatment [*F*_(5, 48)_ = 81860.0, *P* < 2.0 × 10^−16^] (Table 4). Similar patterns were observed in AM + RC + MO + PFA treatment (26.1–4.3 motile stages) [*F*_(5, 48)_ = 83490.0, *P* < 2.0 × 10^−16^], AM + NG + MO + PFA treatment (25.9–4.4 motile stages) [*F*_(5, 48)_ = 94,755, *P* < 2.0 × 10^−16^], AD + CA + MO + PFA treatment (25.9–4.7 motile stages) [*F*_(5, 48)_ = 86,054, *P* < 2.0 × 10^−16^], AD + RC + MO + PFA treatment (26.1–4.9 motile stages) [*F*_(5, 48)_ = 13664.0, *P* < 2.0 × 10^−16^], and AD + NG + MO + PFA treatment (26.0–4.9 motile stages) [*F*_(5, 48)_ = 33898.0, *P* < 2.0 × 10^−16^]. Moderate but still significant reductions were also recorded in single treatments. AD alone reduced motile stages densities from 25.8 to 11.1 motile stages [*F*_(5, 48)_ = 32936.0, *P* < 2.0 × 10^−16^], AM alone from 26.1 to 8.0 motile stages [*F*_(5, 48)_ = 3285.0, *P* < 2.0 × 10^−16^], NG alone from 25.5 to 12.4 motile stages [*F*_(5, 48)_ = 937.3, *P* < 2.0 × 10^−16^], CA alone from 25.6 to 11.6 motile stages [*F*_(5, 48)_ = 106.9, *P* < 2.0 × 10^−16^], and RC alone from 25.7 to 11.8 motile stages [*F*_(5, 48)_ = 86.5, *P* < 2.0 × 10^−16^]. Similarly, mineral oil [*F*_(5, 48)_ = 215.4, *P* < 2.0 × 10^−16^] and potassium salts of fatty acids [*F*_(5, 48)_ = 201.1, *P* < 2.0 × 10^−16^] also showed significant efficacy, with final counts of 9.2 and 9.1 motile stages respectively. In contrast, motile stages in the control (26.6–25.4) and Tween 80 treatment (26.2–23.2) showed only slight, non-significant reductions.

**Table 4:** Effect of different treatments on motile stages (nymphs and adults) of *Eugaster spinulosa* under screehouse conditions (26 ± 2 °C, 60 ± 10% relative humidity, under natural light).

**Treatment**	**Density of eggs (Mean** ±**SE)**					
	**Before treatment**	**Week 1**	**Week 2**	**Week 3**	**Week 4**	**Week 5**
T1	26.6 ± 0.3 Aa	25.1 ± 0.5 Aab	26.0 ± 0.3 Abc	25.6 ± 0.3 Abc	25.6 ± 0.3 Abc	25.4 ± 0.2 Ac
T2	26.2 ± 0.3 Aa	24.3 ± 0.5 Ab	24.4 ± 0.2 Bb	23.9 ± 0.2 Bb	23.7 ± 0.2 Bbc	23.2 ± 0.2 Bc
T3	25.8 ± 0.2 Aa	19.8 ± 0.5 Bb	17.4 ± 0.2 Fc	15.0 ± 0.3 Fd	12.8 ± 0.2 Fe	11.1 ± 0.2 Ff
T4	26.1 ± 0.2 Aa	15.6 ± 0.2 Cb	13.7 ± 0.2 Ic	11.6 ± 0.2 Id	9.7 ± 0.2 Ie	8.0 ± 0.2 If
T5	25.5 ± 0.2 Aa	20.6 ± 0.4 Bb	18.9 ± 0.2 Cc	16.2 ± 0.2 Cd	14.3 ± 0.2 Ce	12.4 ± 0.2 Cf
T6	25.7 ± 0.2 Aa	19.5 ± 1.1 Bb	18.2 ± 0.2 Db	15.6 ± 0.2 Dc	13.8 ± 0.2 Dd	11.8 ± 0.2 De
T7	25.6 ± 0.2 Aa	19.1 ± 1.0 Bb	17.6 ± 0.2 Eb	15.2 ± 0.2 Ec	13.4 ± 0.1 Ed	11.6 ± 0.1 Ee
T8	25.6 ± 0.2 Aa	17.1 ± 0.8 Cb	15.3 ± 0.2 Gc	13.2 ± 0.2 Gd	11.1 ± 0.2 Ge	9.2 ± 0.2 Gf
T9	25.8 ± 0.2 Aa	16.6 ± 0.8 Cb	14.8 ± 0.2 Hc	12.9 ± 0.2 Hd	10.9 ± 0.2 He	9.1 ± 0.1 Hf
T10	26.0 ± 0.2 Aa	10.7 ± 0.7 Db	8.8 ± 0.1 Jc	7.2 ± 0.2 Kd	5.8 ± 0.1 Je	4.9 ± 0.1 Kf
T11	26.1 ± 0.2 Aa	10.9 ± 0.1 Db	8.6 ± 0.1 Kc	7.4 ± 0.2 Jd	5.8 ± 0.1 Ke	4.9 ± 0.1 Jf
T12	25.9 ± 0.2 Aa	10.4 ± 0.3 Db	8.4 ± 0.1 Lc	7.0 ± 0.2 Ld	5.6 ± 0.1 Le	4.7 ± 0.1 Lf
T13	26.0 ± 0.2 Aa	9.9 ± 0.2 Db	7.9 ± 0.2 Mc	6.5 ± 0.1 Md	5.3 ± 0.1 Me	4.4 ± 0.1 Mf
T14	26.1 ± 0.2 Aa	10.1 ± 0.3 Db	7.8 ± 0.2 Oc	6.4 ± 0.2 Nd	5.1 ± 0.1 Oe	4.3 ± 0.1 Of
T15	26.0 ± 0.2 Aa	10.0 ± 0.3 Db	7.9 ± 0.2 Nc	6.5 ± 0.2 Md	5.2 ± 0.1 Ne	4.4 ± 0.1 Nf
Statistical analysis	*F*_14, 120_ = 148.9, *P* = 0.216	*F*_14, 120_ = 92.7, *P* < 2.0 × 10^−16^	*F*_14, 120_ = 97,345.0, *P* < 2.0 × 10^−16^	*F*_14, 120_ = 71512.0, *P* < 2.0 × 10^−16^	*F*_14, 120_ = 106195.0, *P* < 2.0 × 10^−16^	*F*_14, 120_ = 84024.0, *P* < 2.0 × 10^−16^

#### 3.2.3 Effect of treatments on treated plant visual quality

**Figure 2:**
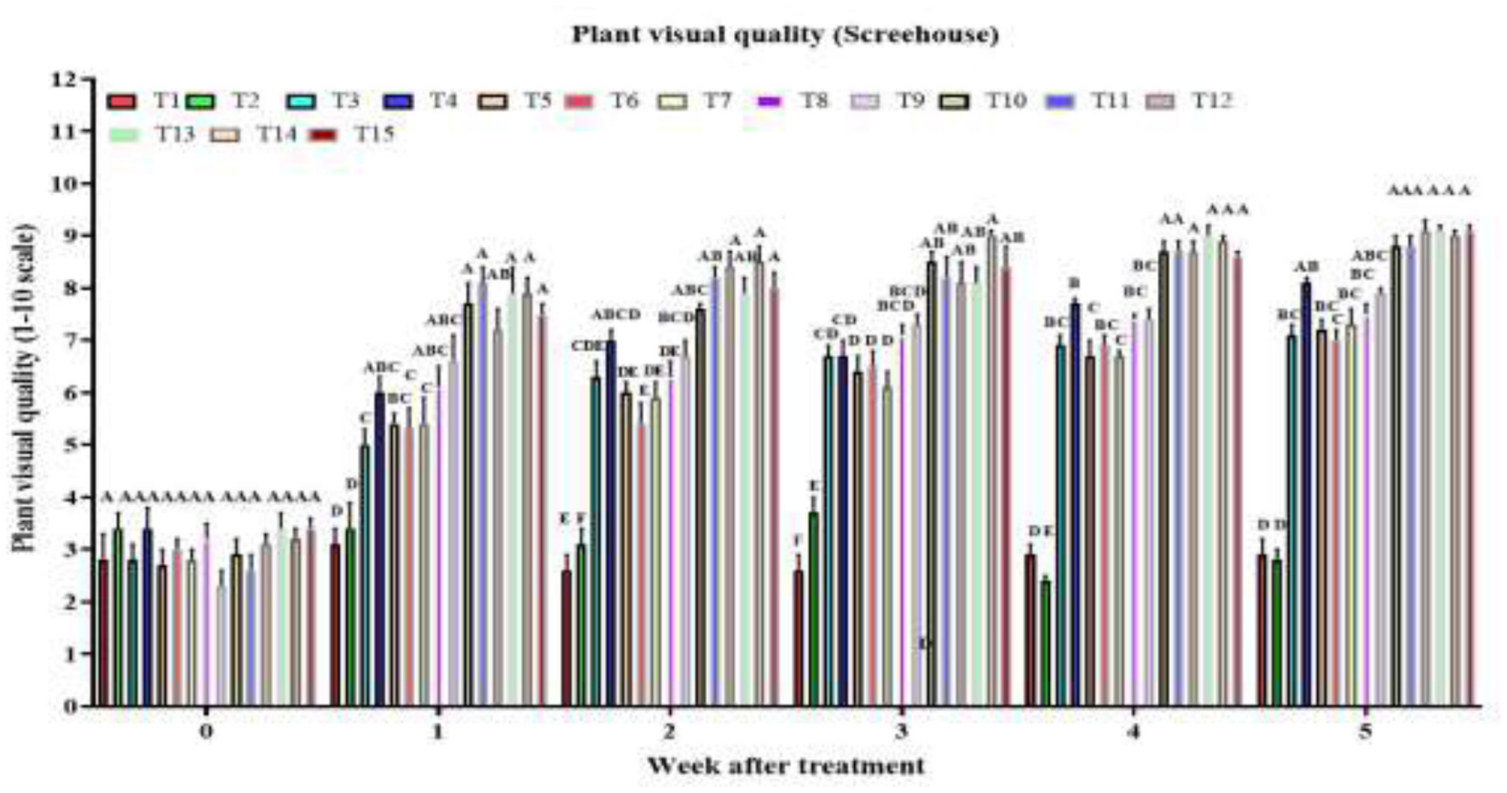
Visual quality of *O. ficus-indica* plants after spray application of untreated control (T1), 0.003% (v/v) Tween 80 (T2), *Alternaria destruens* at 1.0 × 10^10^ conidia/mL alone (T3), *Alternaria murispora* at 1.0 × 10^10^ conidia/mL alone (T4), *Nicotiana glauca* at 10% (T5), *Ricinus communis* at 10% (T6), *Capsicum annuum* at 10% (T7), mineral oil at 1,000 cc/hL (T8), potassium salts of fatty acids at 300 cc/hL (T9), AD + NG + MO + PFA (T10), AD + RC + MO + PFA (T11), AD + CA + MO + PFA (T12), AM + NG + MO + PFA (T13); AM + RC + MO + PFA (T14), and AM + CA + MO + PFA (T15).Visual quality is rated on a scale from 0 to 10, where 0 represents dead; 5 denotes fair quality with acceptable form and color, minimal chlorosis or necrosis; and 10 indicates excellent quality, healthy and robust with optimal color and form. Different letters above bars indicate statistical differences (based on the Student-Newman-Keuls test, α = 0.05). The order of treatments in the figure corresponds exactly to the order listed here, from left to right, to facilitate tracking of each treatment's data.

By week 1, there was a highly significant treatment effect on plant visual quality score [*F*_(14, 120)_ = 16.8, *P* < 2 × 10^−16^], with combined treatments AM + RC + MO + PFA, AM + CA + MO + PFA, AM + NG + MO + PFA, AD + NG + MO + PFA, and AM + CA + MO + PFA showing the highest visual quality scores (Figure 2). In week 2, the treatment effect remained highly significant [*F*_(14, 120)_ = 44.1, *P* < 2 × 10^−16^], with the same combined treatments maintaining superior scores. The strong treatment effect persisted in week 3 [*F*_(14, 120)_ = 44.64, *P* < 2 × 10^−16^], with AM + RC + MO + PFA treatment being the most effective (visual quality score: 9.0). By week 4, the significant effect continued [*F*_(14, 120)_ = 152.7, *P* < 2 × 10^−16^], with AM + NG + MO + PFA, AM + CA + MO + PFA, and AM + RC + MO + PFA treatments achieving the highest visual quality scores. In week 5, the highly significant treatment effect [*F*_(14, 120)_ = 114.6, *P* < 2 × 10^−16^] highlighted the tested combined treatments AM + RC + MO + PFA, AM + NG + MO + PFA, AM + CA + MO + PFA, AD + RC + MO + PFA, AD + NG + MO + PFA, and AD + CA + MO + PFA as the most effective (visual quality score: 8.8-9.1). During the study period (by week 5), the control showed no significant change in visual quality [3.0–3.0; *F*_(5, 48)_ = 0.4, *P* = 0.876], and Tween 80 caused only a slight, non-significant decline [3.0–2.8; *F*_(5, 48)_ = 2.2, *P* = 0.074]. In contrast, all other treatments significantly improved plant visual quality. Single treatments raised scores as follows: AD [to 6.6; *F*_(5, 48)_ = 35.4, *P* = 5.3 × 10^−15^], AM [to 8.2; *F*_(5, 48)_ = 30.6, *P* = 7.7 × 10^−14^], NG [to 7.2; *F*_(5, 48)_ = 40.1, *P* = 5.0 × 10^−16^], RC [to 7.1; *F*_(5, 48)_ = 26.9, *P* = 7.2 × 10^−11^], CA [to 6.7; *F*_(5, 48)_ = 30.0, *P* = 1.1 × 10^−11^], MO [to 7.5; *F*_(5, 48)_ = 33.3, *P* = 1.7 × 10^−14^], and PFA [to 7.9; *F*_(5, 48)_ = 45.5, *P* < 2.0 × 10^−16^]. Combined treatments were most effective, with scores increasing to 9.1–9.3 in all cases: AD + NG + MO + PFA [*F*_(5, 48)_ = 84.6, *P* < 2.0 × 10^−16^], AD + RC + MO + PFA [*F*_(5, 48)_ = 75.6, *P* < 2.0 × 10^−16^], AD + CA + MO + PFA [*F*_(5, 48)_ = 85.5, *P* < 2.0 × 10^−16^], AM + NG + MO + PFA [*F*_(5, 48)_ = 61.7, *P* < 2.0 × 10^−16^], AM + RC + MO + PFA [*F*_(5, 48)_ = 118.8, *P* < 2.0 × 10^−16^], and AM + CA + MO + PFA [*F*_(5, 48)_ = 102.8, *P* < 2.0 × 10^−16^].

## 4 Discussion

This study confirms the effectiveness of integrated biorational and biological treatments for managing *E. spinulosa* on *Opuntia* spp. Laboratory and screenhouse trials showed that combinations of *A. murispora* or *A. destruens* with *C. annuum, R. communis*, or *N. glauca* extracts, along with mineral oil and potassium salts of fatty acids, outperformed individual treatments in reducing egg-laying, suppressing motile stages, and enhancing plant visual quality. Treatment efficacy varied significantly over time, highlighting the importance of both treatment type and exposure duration.

Among the single treatments, AM and AD applied independently demonstrated moderate efficacy, with AM consistently outperforming AD across all observation periods. This finding aligns with previous studies reporting the higher virulence and faster action of *A. murispora* (El Aalaoui et al., 2024a). The entomopathogenic properties of *Alternaria* spp. have been documented against a wide range of pests affecting various crops (Sharma and Sharma, 2021). In *Opuntia* spp. cultivation, both *Alternaria* spp. have induced significant mortality in *D. opuntiae* nymphs, with reported rates ranging from 70% to 90% under laboratory conditions (Ouguas et al., 2022). Furthermore, *A. murispora* (PP264308) and *A. destruens* (PP264311), were effective against *D. opuntiae* adult females under field conditions, achieving 84.2% and 75.8% mortality, respectively, at a concentration of 108 conidia/mL, 10 days post-application (El Aalaoui et al., 2024a). This strong pathogenic activity is consistent with the known entomopathogenic properties of *Alternaria* spp., which are capable of producing diverse bioactive compounds, including destruxins and chitinases that contribute to insect mortality (Green et al., 2001; Chen et al., 2021). However, since *A. alternata* (Fr.) Keissl (Pleosporales: Pleosporaceae) and related species are known to produce more than 70 toxins, including mycotoxins that may pose risks to non-target organisms, further evaluation is required to ensure the safety and suitability of *A. murispora* for agricultural application (Chen et al., 2021). In addition, integrating *A. murispora* with *Chilocorus bipustulatus* Linnaeus and *Exochomus nigripennis* (Erichson) (Coleoptera: Coccinellidae) has significantly reduced *D. echinocacti* infestations on *O. ficus-indica* without negatively affecting plant quality (El Aalaoui et al., 2024b). *Nicotiana glauca* (NG) has shown insecticidal properties against several pest species, including *D. opuntiae* (Zim et al., 2025), *P. solenopsis* (Abdelgader and Elawad, 2022), and the Red Palm Weevil *Rhynchophorus ferrugineus* (Olivier) (Coleoptera: Curculionidae) (Alghamdi, 2021). *Capsicum annuum* has also demonstrated efficacy against various insect including mosquito larvae such as *Culex quinquefasciatus* Say and *Anopheles stephensi* L. (Diptera: Culicidae) (Madhumathy et al., 2007), *Sitotroga cerealella* (Olivier) (Lepidoptera: Gelechiidae) (Prakash and Rao, 2006), and the stored-product beetles including *Sitophilus zeamais* Motschulsky (Coleoptera: Curculionidae) and *Tribolium castaneum* (Herbst) (Coleoptera: Tenebrionidae) (Ho et al., 1997), and *Earias insulana* (Boisd.) (Lepidoptera: Nolidae) (Weissenberg et al., 1986). *Ricinus communis* has shown insecticidal effects against several pests, including *D. opuntiae* (Da Silva Santos et al., 2016; Ramdani et al., 2021), *Aedes aegypti* (Linnaeus) (Diptera: Culicidae) (Neves et al., 2014), and *Spodoptera frugiperda* (J.E. Smith) (Lepidoptera: Noctuidae) (Ramos-López et al., 2010). Among these botanical extracts evaluated, CA and RC exhibited stronger insecticidal activity than NG. This suggests that capsaicinoids—the primary bioactive compounds in CA (Chilczuk et al., 2021)—as well as ricin (a toxic protein) and ricinine (an alkaloid) in RC (Elijah and Somadina, 2020), may be responsible for the observed potency. However, while ricin and ricinine enhance the insecticidal efficacy of RC, both compounds have known toxicity concerns. Ricin is an extremely potent ribosome-inactivating protein that can cause severe cytotoxicity even at low doses (Stirpe, 2013), while ricinine, though less toxic, has been reported to induce neurotoxic and hepatotoxic effects in mammals (Audi et al., 2005). These toxicological risks highlight the importance of assessing the safety profile of RC extracts before field application. Therefore, future studies should aim to determine safe and effective concentrations, evaluate formulations that reduce mammalian toxicity, and ensure that the use of RC-based biopesticides poses no risk to humans, beneficial insects, or the environment. Furthermore, the insecticidal potency of plant extracts can vary based on the arthropod species and the specific plant organ used, as the concentration of active compounds differs among plant parts (Costa et al., 2004; Santiago et al., 2008). Similarly, mineral oil and potassium salts of fatty acids as standalone treatments provided moderate suppression, likely due to their mode of action involving disruption of the insect cuticle and respiratory system (Helmy et al., 2012). Their insecticidal activity against pests like aphids, thrips, and mites has been confirmed in several studies, especially at concentrations up to 2% with repeated use (Wins-Purdya et al., 2009; de Souza et al., 2009; Curkovic, 2016). However, when comparing treatment types, it is evident that EPF-based treatments (T3–T4) had generally higher and more consistent impact than botanical extracts alone (T5–T7), particularly beyond week 2. This supports the idea that fungi, once established, continue to infect hosts, while plant extracts tend to degrade or lose efficacy over time due to environmental factors such as light or temperature (Inglis et al., 1997).

The most effective treatments under both laboratory and screenhouse conditions were AM + CA + MO + PFA, AM + RC + MO + PFA, and AM + NG + MO + PFA. These combinations consistently reduced egg and motile stage densities by over 80% by week 5 post-treatment, with significant differences evident from the first week. The high efficacy observed suggests a synergistic interaction between the entomopathogenic fungus *A. murispora*, botanical extracts, and biorational insecticides. Each component likely contributes through distinct yet complementary mechanisms: MO and PFA disrupt the insect cuticle and impede respiration, plant extracts exert antifeedant or repellent effects, and the fungus induces pathogenic infection. Previous studies have shown that vegetable oils, plant extracts, and soaps enhance the effectiveness of fungal pathogens, promoting the use of oil-based formulations in pest management (Wraight et al., 2016; Lei et al., 2023). These substances spread rapidly across the insect cuticle, helping conidia access protected areas such as intersegmental membranes, where higher humidity can support fungal germination and infection (Luz et al., 2012). Since conidia are essential for initiating infection and maintaining fungal viability under field conditions, preserving their germination potential is critical for the success of integrated pest management (Da Silva Santos et al., 2016).

Comparing fungal combinations, those containing *A. murispora* (T13–T15) consistently outperformed those with *A. destruens* (T10–T12), indicating a higher pathogenic potential of *A. murispora* under varied conditions. This may be linked to faster conidial germination, stronger adhesion to the host cuticle, or greater enzymatic activity (El Aalaoui et al., 2024a). Regarding plant extracts, combinations with CA and RC were more effective than those with NG, underscoring the importance of selecting botanicals with potent contact or systemic properties. Differences in efficacy may be attributed to variations in their chemical constituents, such as alkaloids, phenolic compounds, and terpenoids. The progressive decline in pest densities observed over the 5-week period highlights the importance of sustained treatment effects, particularly in managing pest populations with overlapping life stages like *E. spinulosa*. This temporal dynamic aligns with prior studies showing cumulative impacts of entomopathogenic fungi and low-toxicity insecticides under semi-field conditions (Inglis et al., 2001; Sevim et al., 2013). While all single treatments showed moderate efficacy, their performance was consistently inferior to that of the integrated approaches. Although AM alone reduced egg numbers significantly (up to 57% in lab conditions), its efficacy was markedly enhanced when combined with MO, PFA, and plant extracts—suggesting that cuticle disruption and increased contact/adhesion may enhance fungal infection.

Importantly, plant visual quality assessments revealed parallel improvements in treated plants, with the highest scores recorded in the same combination treatments that suppressed *E. spinulosa* most effectively. This result not only supports the pest suppression data but also confirms the phytocompatibility and potential crop-sparing benefits of these treatments. The visual scale scores increased from ~3.0 before treatment to over 9.0 in the best-performing treatments, indicating effective mitigation of pest-induced stress and physical damage. The results from screenhouse trials largely mirrored those from the laboratory, affirming the robustness of the treatments under more variable environmental conditions. However, the slightly lower efficacy of AD-based combinations compared to AM-based ones under screenhouse conditions may reflect differences in fungal persistence or infectivity in semi-natural microclimates. This observation aligns with previous studies reporting differential conidial stability and environmental sensitivity among fungal strains (Rangel et al., 2015; Lima et al., 2021; Wang et al., 2021).

## 5 Conclusions

The integration of *A. murispora* with plant extracts from *C. annuum, R. communis, N. glauca*, mineral oil, and potassium salts of fatty acids significantly reduced *E. spinulosa* egg and motile stage densities in lab and screenhouse trials. Combinations with *C. annuum* and *R. communis* showed the best results, underscoring the value of compatible botanicals in IPM. These multi-component formulations, combining fungal, botanical, and biorational insecticides, offer promising sustainable pest control. Future work should confirm these effects in open fields and evaluate ecological impacts.

## Data Availability

The original contributions presented in the study are included in the article/supplementary material, further inquiries can be directed to the corresponding author.
